# Blood pressure change across pregnancy in white British and Pakistani women: analysis of data from the Born in Bradford cohort

**DOI:** 10.1038/s41598-019-49722-9

**Published:** 2019-09-13

**Authors:** Diane Farrar, Gillian Santorelli, Debbie A. Lawlor, Derek Tuffnell, Trevor A. Sheldon, Jane West, Corrie Macdonald-Wallis

**Affiliations:** 1 Bradford Institute for Health Research, Bradford Royal Infirmary, Bradford, UK; 2 MRC Integrative Epidemiology Unit, University of Bristol, Bristol, UK; 3 Population Health Science, Bristol Medical School, University of Bristol, Bristol, UK; 4 Bristol National Institute for Health Research Biomedical Resource Centre, Bristol, UK; 5 Bradford Women’s and Newborn Unit, Bradford, UK; 6 Department of Health Sciences, University of York, York, UK; 7 Centre for Exercise, Nutrition and Health Sciences, University of Bristol, Bristol, UK

**Keywords:** Pre-eclampsia, Pre-eclampsia

## Abstract

The incidence of gestational hypertension (GH) and pre-eclampsia (PE) is increasing. Use of blood pressure (BP) change patterns may improve early detection of BP abnormalities. We used Linear spline random-effects models to estimate BP patterns across pregnancy for white British and Pakistani women. Pakistani women compared to white British women had lower BP during the first two trimesters of pregnancy, irrespective of the development of GH or PE or presence of a risk factor. Pakistani compared to white British women with GH and PE showed steeper BP increases towards the end of pregnancy. Pakistani women were half as likely to develop GH, but as likely to develop PE than white British women. To conclude; BP trajectories differ by ethnicity. Because GH developed evenly from 20 weeks gestation, and PE occurred more commonly after 36 weeks in both ethnic groups, the lower BP up to the third trimester in Pakistani women resulted in a lower GH rate, whereas PE rates, influenced by the steep third trimester BP increase were similar. Criteria for diagnosing GH and PE may benefit from considering ethnic differences in BP change across pregnancy.

## Introduction

Ten percent of pregnant women will develop gestational hypertension (GH) or pre-eclampsia (PE)^1–4^. The incidence of these conditions is increasing and influenced by population characteristics, in particular the rise in overweight and obesity^5,6^. GH and PE are major causes of maternal and infant perinatal morbidity and mortality^7–9^. There is also growing evidence that the longer-term cardiovascular health of the mother and offspring exposed to GH or PE may be adversely affected^10,11^. Consequently the burden to the individual and to health services is growing^12–14^. Early detection and treatment of pregnancy hypertension can reduce risks^15–17^, but this early detection is somewhat hampered by the diagnosis of GH or PE being defined as hypertension after 20 weeks of gestation (i.e. after the nadir of early pregnancy decline in BP reported in many previous studies)^18–20^.

Hypertensive disorders of pregnancy (HDP) which includes GH and PE, represents a spectrum of disorders, all of which are characterised by high diastolic (DBP) or systolic (SBP) blood pressure (BP)^21^. Elevated BP after 20 weeks in women without a history of hypertension is defined as GH; when it occurs with clinically significant levels of proteinuria it is PE^21,22^. The offspring of women who develop PE, more so than those developing GH, are at greater risk of adverse outcomes including preterm birth and small for gestational age^23^. Normal pregnancy is characterised by decreasing levels of BP from conception to 20 weeks then a slight and steady increase thereafter to term^20^. Elevated BP after 20 weeks in all cases of HDP can be reached by different patterns of gestational BP change, including higher than average levels from conception, a smaller decline in BP in early pregnancy and/or a more rapid or pronounced rise following this early pregnancy decline. Whether different patterns of reaching the criteria for GH and PE are helpful in predicting more adverse outcomes, or what risk factors influence them is unclear and this information may be important when considering diagnostic thresholds and treatment of GH and PE in different ethnic groups.

We have shown previously that in white European women without GH or PE, mean BP at the start of pregnancy, the rate of decline in early pregnancy and the steepness of the increase in late pregnancy, differs by established risk factors (age, parity, BMI, multiple pregnancy) in the same direction that those risk factors are associated with HDP^18,24^. We have further shown that early BP change can be used to predict which women are likely to experience a HDP, and proposed gestational BP centile charts for use in white European women^25,26^.

South Asians, in comparison to white Europeans, have higher levels of adiposity for a given body mass from birth, and are at increased risk of insulin resistance, diabetes and coronary heart disease^27–30^. In pregnancy this is reflected in higher rates of gestational diabetes and mean levels of fasting and post-load glucose^29,31^. Greater adiposity and gestational diabetes are both associated with an increased risk of a HDP^23,32,33^, therefore we might expect HDP to be more common in south Asian women. However, the small number of studies that have compared the incidence of HDP between south Asian and white Europeans have found either a lower or similar incidence^34–37^. To our knowledge few previous studies have examined ethnic differences in SBP and DBP change across pregnancy. Those that have include few women from ethnic groups other than white European^38,39^.

The aims of this study were to explore whether: (i) patterns of BP change in pregnancy differed between white British and Pakistani women; (ii) any differences in BP change between these two ethnic groups were related to differences in GH and PE incidence and whether (iii) established risk factors (maternal age, BMI and parity) for GH and PE differed between the ethnic groups in how they relate to BP trajectories and GH and PE incidence.

By addressing these aims we hope to better understand the mechanisms that might lead to ethnic differences in HDP incidence and to provide the foundations for exploring whether different thresholds for diagnosing GH and PE between these different ethnic groups would improve their detection, management and outcomes.

## Methods

### Participants

**Figure 1:**
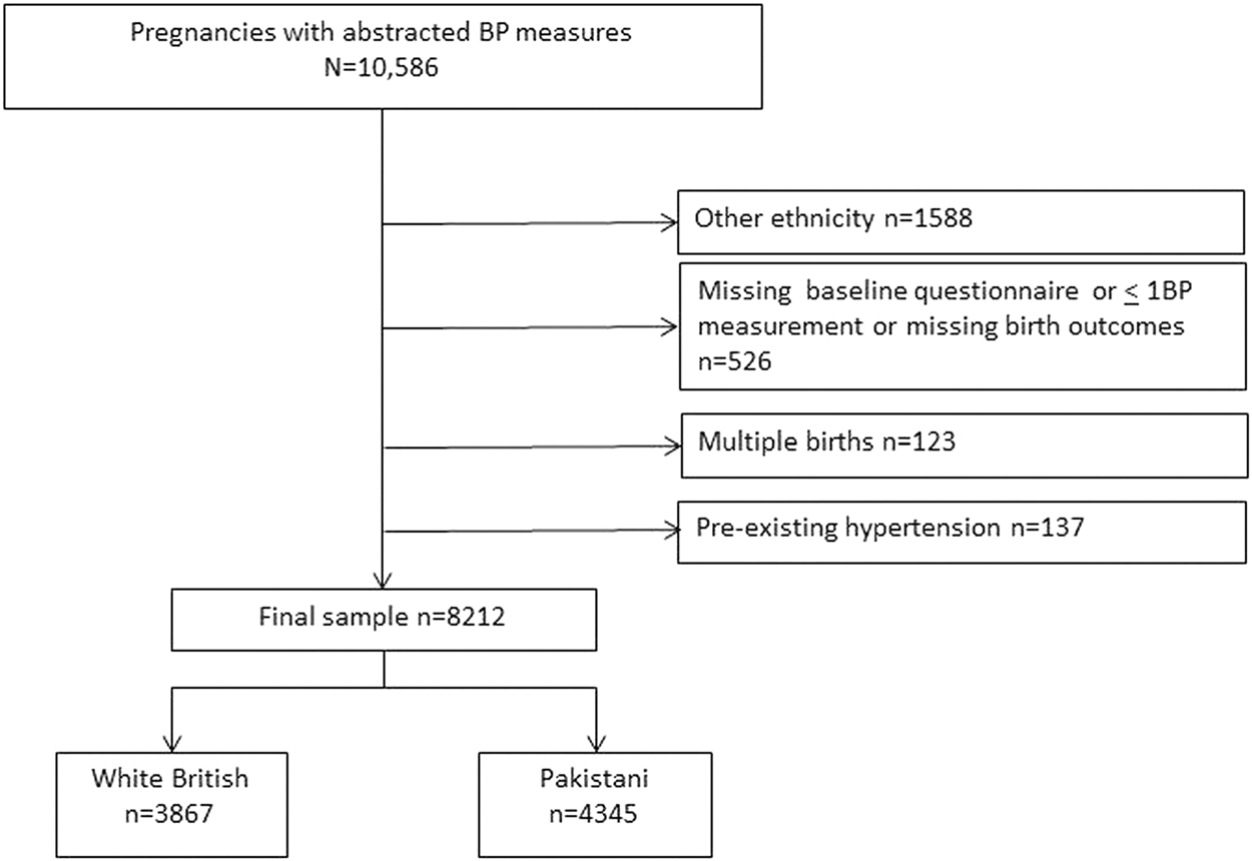
Study flow chart.

Our analyses were undertaken in two ethnic groups – white British and Pakistani women, because these represent the largest ethnic groups in our cohort study. Data from women participating in the Born in Bradford (BiB) cohort were used. BiB is a prospective birth cohort study including 12,450 women who booked to deliver at the Bradford Royal Infirmary, Bradford, UK, between 2007 and 2011. The cohort is broadly representative of the obstetric population in Bradford and includes equal numbers of Pakistani and white British women. The study methods have been described in full elsewhere^40^. At recruitment, participants gave informed written consent, height and weight was measured, a questionnaire was administered by trained research assistants and information was abstracted from medical records, informed written consent was also given by participants for the future abstraction of information from medical records. Interviews were conducted in English or in south Asian languages (including Urdu and Mirpuri). Research Ethics Committee approval was obtained (07/H1302/112), all research was performed in accordance with relevant guidelines and regulations. Women were excluded if they did not complete a baseline questionnaire, had one or less BP measurement recorded antenatally or did not have their birth outcomes recorded. Following further exclusions (women with pre-existing hypertension, multiple pregnancies or ‘other’ ethnicities (than white British or Pakistani)), 8212 women were included in these analyses. Figure 1 shows the full inclusion and exclusion criteria of women from BiB for this study.

### Outcomes

All BP and proteinuria measurements that were recorded as part of routine antenatal care were abstracted from each participant’s medical records (i.e. each woman would have several repeat measurements of SBP, DBP and proteinuria).

These repeat measurements were conducted by midwives, doctors or health care assistants working in the community or hospital clinics, who were trained to measure BP in the seated position using the appropriate cuff size, diastolic BP (DBP) was measured using Korotkoff phase V. Most measurements were scheduled according to UK clinical guideline recommendations (at 10, 16, 28, 34, 36, 38, 41 weeks gestation and additionally at 25, 31 and 40 weeks gestation for women during their first pregnancy)^41^. Women who were deemed to require additional monitoring by their clinical team or who self-referred for various reasons (for example abdominal pain or reduced fetal movements) had measurements recorded at additional times. A variety of devices were used to measure BP and proteinuria, all devices were calibrated according to standard UK health service practice^21^. The gestational age on the date of each measurement was derived from the infant’s gestational age at birth and date of measurement, gestational age at birth was derived from the 10 week ultrasound scan and date of birth.

The extraction of BP and proteinuria measurements from paper medical records was undertaken by four trained research assistants. Accuracy of abstractions was checked on 5% of records for all abstracted data; all error rates were less than 1%. The BP and proteinuria repeat measurements were used to derive gestational BP change trajectories across pregnancy and also to define GH and PE in a systematic way for all participants. GH was defined as new-onset hypertension after 20 weeks gestation; DBP equal to or exceeding 90 mmHg and/or SBP equal to or exceeding 140 mmHg on at least two separate occasions. PE was defined as for GH, but also with concurrent (with the elevated blood pressure) clinically significant proteinuria; greater or equal to 1 ‘+’ on the reagent strip reading (the equivalent of ~30 mg/mmol [0.3 mg/mg] on spot urine protein/creatinine ratio)^21,22^.

### Ethnicity, HDP risk factors and other measurements

The woman’s ethnicity, age, education, parity and smoking during pregnancy were obtained from interviewer administered questionnaires at recruitment. Ethnicity was categorised as white British and Pakistani according to UK Office of National Statistics criteria^42^. Education was equivalized to UK standard attainments and participants were included in one of five mutually exclusive categories (<5 GCSE equivalent, +5 GCSE equivalent, A level equivalent, higher, other)^29^. Family history of diabetes and hypertension was abstracted from antenatal clinical paper records as described above for the repeat BP and proteinuria measures. Maternal weight as recorded at the first antenatal clinic visit, glucose levels (at the 26–28 week gestation oral glucose tolerance test) and infant sex^43^ were obtained from hospital electronic records. Gestational diabetes was defined according to modified World Health Organization (WHO) criteria operating at the time of recruitment (either fasting glucose ≥6·1 mmol/L, or 2 h post-load glucose ≥7·8 mmol/L)^29,44^.

Body mass index (BMI kg/m2) was calculated using height from the recruitment assessment and the first antenatal clinic visit weight, which was generally before 12 weeks gestation, as that would be little influenced by pregnancy or fetal size in comparison to the measure at recruitment (around 28 weeks). BMI was categorised according to WHO definitions of underweight or normal (≤24.9 kg/m^2^; there being too few women to categorize underweight separately), overweight (≥25.0–29.9 kg/m^2^) and obese (≥30.0 kg/m^2^)^45^. Smoking during pregnancy which was self-reported and categorized as yes or no.

### Statistical analysis

Linear spline random-effects models were used to derive SBP and DBP change trajectories across gestation, as previously described^18,24^. Multilevel modelling (using antenatal visit as level 1 and woman as level 2) was conducted to account for multiple antenatal measurements and clustering (non-independence) of these within each woman. The median, interquartile range, and full range of the number of BP measurements per woman were 10, 8–14 and 1–142 respectively. In order to prevent the small number of women with many repeat measurements overly influencing results we split gestation into two week periods and for any woman who had more than one measure in any of these two-week periods, one measurement was selected at random to remain in the analyses. This was achieved by creating a random number for each record using the invnorm(uniform)) command in Stata, sorting data by participant identifier, gestation period and random number, then choosing the first record within each gestation period. Following this, there was a median of 9 BP measurements per woman (interquartile range, 8–10 and full range 2–16).

The best fitting model was derived using fractional polynomials; we examined these in the whole cohort and for the two ethnic groups, with the appearance of the average cohort trajectory from this modelling used to explore potential knot points. Knot points are the gestational ages at which a change in the direction or magnitude of BP trajectory slopes occurs. We examined the five highest log-likelihoods for SBP and DBP models separately, in the whole cohort and also separately for white British and Pakistani women. Our aim was to identify knot points that were a good reflection (fit) of the observed data and if possible have similar knot points for both SBP and DBP and for both ethnic groups in order to aid comparisons. We set eight weeks gestation as the baseline (intercept) as there were too few measurements before that time to produce meaningful results. The modelling created five measurements of SBP and DBP change for each woman: baseline level at 8 weeks (intercept) and change between 8 and 24, 24 and 30, 30 and 36 weeks and 36 weeks to delivery.

Women with PE have a high risk of preterm delivery, particularly if maternal BP rises rapidly, and earlier delivery may lead to fewer measurements contributing to the models and thus a lower influence from pregnancies with a steeper increase in BP, so underestimating the slope. In order to adust for this, we combined the random-effects spline models for BP change with length of gestation to produce a joint model^18^. Individual trajectories and trajectories by categorical covariates were obtained from random-intercept and -slope models, which provide a measure of each individual’s deviation from the whole cohort mean BP at 8-weeks and from the average slope in each period of gestation. The following covariates were explored in these models: HDP (normotensive (reference group), GH, PE); age (<25 (reference) 25–29, 30–35, >35); BMI (underweight/normal (reference), overweight, obese); ethnicity (white British (reference), Pakistani); parity (nulliparous (reference), multiparous); smoking in pregnancy (no (reference), yes); gestational diabetes (no (reference), yes); education (<5 GCSE equivalent (reference), 5+ GCSE equivalent, A level equivalent, higher, other) and offspring sex (male (reference), female).

The associations between BP changes and time to delivery were derived from the random effects for BP outcomes and the residual error from the length of gestation. Our initial model (model 1) adjusted for all covariates (described above). In model 2, we additionally adjusted each slope (mean change in BP per gestational week) for the mean BP at 8 weeks, and each proceeding slope(s), so that results were not confounded by the initial BP measure and earlier slope(s) which correlate with subsequent slopes^18,24^.

### Ethics approval

Ethics approval was granted by the Bradford National Health Service Research Ethics Committee (ref 07/H1302/112) on 10 March 2008.

## Results

Table 1 shows the distribution of characteristics of all women and by ethnic group; 4345 were Pakistani and 3867 white British. Compared to white British women, Pakistani women were older, were more likely to have attained a higher educational level, to be multiparous, less likely to be obese or to have smoked in pregnancy and more likely to have developed gestational diabetes in the current pregnancy and to have a family history of diabetes or hypertension.

**Table 1:** Characteristics for all participants and by ethnicity.

	All women N = 8,212	White British women N = 3,867	Pakistani women N = 4,345	P-value^c^
Gestational age at delivery, weeks^a^	39 (38–40)	40 (39–40)	39 (38–40)	<0.001
**Hypertensive disorder of pregnancy (HDP)**				
No HDP	7,376 (90)	3,326 (86)	4,050 (93)	<0.001
Gestational hypertension	622 (8)	428 (11)	194 (5)	
Pre-eclampsia	214 (3)	113 (3)	101 (2)	
**Maternal age at infant birth (years)**				
<25	2,788 (34)	1,516 (39)	1,272 (29)	<0.001
25–29	2,641 (32)	1,090 (28)	1,551 (36)	
30–34	1,778 (22)	767 (20)	1,011 (23)	
≥35	1,005 (12)	494 (13)	511 (12)	
BMI at pregnancy booking (kg/m^2^)^b^ (mean)	26.2	26.9	25.6	<0.001
Underweight (BMI ≤ 19)	503 (6)	154 (4)	349 (8)	
Normal (BMI 19–24)	3,482 (42)	1,608 (42)	1,874 (43)	
Overweight (BMI 25–29)	2,446 (30)	1,126 (29)	1,320 (30)	
Obese (BMI > 30)	1,781 (22)	979 (25)	802 (19)	
**Parity:**				
Nulliparous	3,291 (40)	1,875 (49)	1,416 (33)	<0.001
Multiparous	4,921 (60)	1,992 (52)	2,929 (67)	
**Smoked during pregnancy**				
Yes	1,452 (18)	1,302 (34)	150 (4)	<0.001
**Highest educational achievement**				
<5 GCSE equivalent	1,883 (23)	766 (20)	1,117 (26)	<0.001
5+ GCSE equivalent	2,694 (33)	1,332 (35)	1,362 (31)	
A-level equivalent	1,221 (15)	659 (17)	562 (13)	
Higher	1,867 (23)	744 (19)	1,123 (26)	
Other	547 (7)	366 (10)	181 (4)	
**Infant gender**				
Male	4,212 (51)	1,990 (52)	2,222 (51)	0.771
Female	4,000 (49)	1,877 (49)	2,123 (49)	
Gestational diabetes	660 (8)	191 (5)	469 (11)	<0.001
Family history of diabetes	2,045 (25)	514 (13)	1,531 (35)	<0.001
Family history of hypertension	2,167 (26)	901 (23)	1,266 (29)	<0.001
Pre-existing diabetes	15 (<1)	9 (<1)	6 (<1)	0.316

Values are n (%) unless otherwise indicated.

^a^Median and interquartile range.

^b^Body mass index.

^c^Difference between white British and Pakistani women using Wilcoxon rank sum test or chi-squared test as appropriate.

Approximately twice as many white British than Pakistani women developed GH (11% vs 5%), whereas similar proportions of white British and Pakistani women developed PE (3% vs 2%). Of those who developed GH in both ethnic groups approximately half developed it between 20 to 36 weeks of gestation and half at or after 36 weeks, whereas most cases of PE in both groups developed at or after 36 weeks (see Table 2).

**Table 2:** Total numbers developing gestational hypertension at any time in pregnancy: 428 White British and 194 Pakistani women; Total numbers developing preeclampsia at any time in pregnancy:113 White British and 101 Pakistani women.

Hypertensive disorder of pregnancy	Number of weeks of gestation at which each hypertensive disorder of pregnancy developed	
	20–36 weeks	≥36
**Gestational hypertension N (%)**		
White British	231 (54)	197 (46)
Pakistani	86 (44)	108 (56)
**Preeclampsia N (%)**		
White British	15 (13)	98 (87)
Pakistani	25 (25)	76 (75)

Table 3 shows the unadjusted associations between risk factors and GH and PE by ethnicity. Generally, point estimates tended to be closer to the null for Pakistani women than for white British women.

**Table 3:** Unadjusted associations of risk factors with hypertensive disorders of pregnancy.

	Difference in means (continuous variables), Odds ratios (binary) or relative risk ratios (multinomial) [95% CI]					
	White British women			Pakistani women		
	No HDP (N = 3326) Reference	Gestational hypertension (N = 428)	Preeclampsia (N = 113)	No HDP (N = 4050) Reference	Gestational hypertension (N = 194)	Preeclampsia (N = 101)
**Risk factor**						
BMI (km/m^2^, reference <25)						
25 to 29	1	2.15 (1.61, 2.86)	2.01 (1.24, 3.26)	1	1.35 (0.93, 1.96)	1.61 (0.97, 2.66)
30+	1	5.59 (4.31, 7.24)	3.34 (2.10, 5.32)	1	3.56 (2.53, 5.01)	3.77 (2.34, 6.06)
Maternal age (years, reference <25)						
25 to 29	1	1.22 (0.94, 1.58)	1.01 (0.64, 1.60)	1	1.02 (0.71, 1.48)	0.73 (0.45, 1.20)
30 to 34	1	1.30 (0.97, 1.72)	0.94 (0.55, 1.60)	1	1.08 (0.73, 1.59)	0.67 (0.39, 1.16)
35+	1	2.21 (1.65, 2.96)	1.14 (0.63, 2.06)	1	1.32 (0.84, 2.08)	0.92 (0.49, 1.74)
Parity (≥1 versus 0)	1	0.77 (0.63, 0.94)	0.55 (0.38, 0.81)	1	0.54 (0.40, 0.72)	0.40 (0.27, 0.59)
Gestational diabetes (Yes vs No)	1	1.48 (0.98, 2.22)	1.14 (0.49, 2.63)	1	1.12 (0.72, 1.75)	1.02 (0.54, 1.91)
**Maternal education (reference 5 GCSE)**						
<5 GCSE	1	0.88 (0.65, 1.20)	1.09 (0.66, 1.80)	1	0.96 (0.66, 1.40)	0.76 (0.46, 1.28)
A level	1	1.37 (1.02, 1.83)	0.76 (0.42, 1.39)	1	0.83 (0.51, 1.36)	0.56 (0.27, 1.17)
Degree	1	1.45 (1.10, 1.92)	1.09 (0.66, 1.83)	1	0.93 (0.64, 1.37)	0.79 (0.47, 1.32)
Other	1	1.39 (0.97, 1.98)	0.64 (0.28, 1.44)	1	1.21 (0.61, 2.40)	1.00 (0.39, 2.58)
Smoking in pregnancy (Yes vs No)	1	0.55 (0.44, 0.70)	0.63 (0.41, 0.97)	1	0.72 (0.29, 1.78)	0.55 (0.13, 2.26)
Infant gender (Female vs Male)	1	0.88 (0.72, 1.07)	0.77 (0.52, 1.12)	1	0.86 (0.65, 1.16)	1.20 (0.81, 1.78)

**Figure 2:**
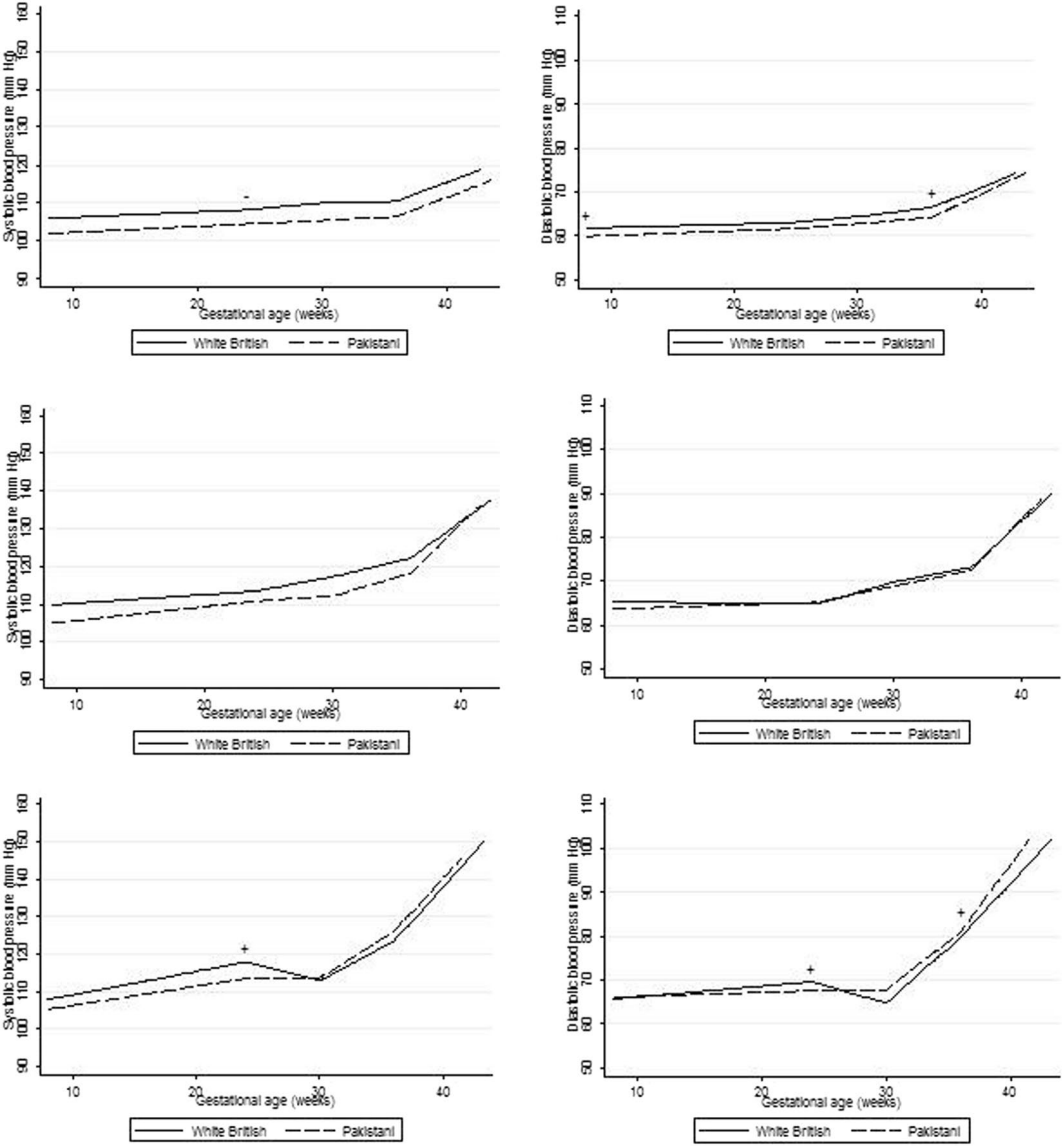
(**a**,**b**) Predicted trajectories of systolic blood and diastolic pressure across pregnancy for Pakistani and white British normotensive women. (**c**,**d**) Predicted trajectories of systolic and diastolic blood pressure across pregnancy for Pakistani and white British women with gestational hypertension. (**e**,**f**) Predicted trajectories of systolic and diastolic blood pressure across pregnancy for Pakistani and white British women with pre-eclampsia. Footnote to (**a** to **f**). − or + Denotes where the mean difference in average BP change in the associated time period between White British and Pakistani women is significantly smaller or larger respectively.

Figure 2a–f show the time to delivery adjusted trajectories (i.e. joint model) of SBP and DBP across pregnancy for Pakistani and white British women who were normotensive (see Fig. 2a,b), and who developed GH (see Fig. 2c,d) or PE (see Fig. 2e,f). Tables 4 and 5 show the corresponding mean differences in SBP and DBP respectively at 8 weeks and the change in BP at each subsequent period. Pakistani women who remained normotensive started pregnancy with lower SBP (−4.00, 95% CI: −4.71 to −3.29) and DBP (−1.92, 95% CI:−2.44 to −1.40) and demonstrated lower BP trajectories across pregnancy compared to white British women.

**Table 4:** Mean difference (95% CI) in SBP at 8 weeks and change in SBP in each period of gestation for each hypertensive disorders of pregnancy.

Hypertensive disorder	Mean difference in average SBP change, mm Hg/wk				
	Mean Difference in SBP at 8 wk, mmHg	8–24 weeks	24–30 weeks	30–36 weeks	≥36 weeks
***No hypertensive disorder***					
White British women	0	0	0	0	0
Pakistani women	−4.00 (−4.71 to −3.29)	0.02 (−0.04 to 0.08)	−0.15 (−0.27 to −0.03)	0.09 (−0.02 to 0.21)	0.01 (−0.19 to 0.20)
***Gestational hypertension***					
White British women	0	0	0	0	0
Pakistani women	−4.93 (−7.74 to −2.12)	0.15 (−0.09 to 0.38)	−0.40 (−0.91 to 0.11)	−0.15 (−0.37 to 0.67)	0.89 (0.03 to 1.74)
***Pre-eclampsia***					
White British women	0	0	0	0	0
Pakistani women	−2.58 (−7.16 to 2.00)	−0.11 (−0.50 to 0.27)	0.84 (0.59 to 1.62)	0.32 (−0.63 to 1.27)	−0.06 (−1.89 to 1.77)

Adjusted model adjusted for time to delivery and for maternal age, pregnancy booking BMI, parity, smoking in pregnancy status, education, gestational diabetes and infant gender.

In reference category: No hypertensive disorder (white British women) mean SBP at 8 wk (mm Hg) 105.84 (104.32 to 107.37); mean SBP change (mm Hg/wk): 8–24 wk 0.15 (0.03 to 0.28); 24–30 wk 0.20 (−0.05 to 0.45); 30–36 wk 0.13 (−0.12 to 0.38); >36 wk 1.33 (0.93 to 1.73).

In reference category: gestational hypertension (white British women), mean SBP at 8 wk (mm Hg) 108.59 (102.95 to 114.23); mean SBP change (mm Hg/wk): 8–24 wk 0.29 (−0.18 to 0.76); 24–30 wk 0.57 (−0.43 to 1.56); 30–36 wk 0.47 (−0.54 to 1.48); >36 wk 3.25 (1.74 to 4.76).

In reference category: pre-eclampsia (white British women), mean SBP at 8 wk (mm Hg) 112.27 (102.17 to 122.37); mean SBP change (mm Hg/wk): 8–24 wk 0.09 (−0.83 to 1.00); 24–30 wk 0.47.

(−1.31 to 2.24); 30–36 wk 1.58 (−0.16 to 3.32); >36 wk 1.13 (−2.25 to 4.52).

**Table 5:** Mean difference (95% CI) in DBP at 8 weeks and change in DBP in each period of gestation for each hypertensive disorders of pregnancy.

Hypertensive disorder	Mean difference in average DBP change, mmHg/wk				
	Mean Difference in SBP at 8 wk, mmHg	8–24 weeks	24–30 weeks	30–36 weeks	≥36 weeks
***No hypertensive disorder***					
White British women	0	0	0	0	0
Pakistani women	−1.92 (−2.44 to −1.40)	0.04 (0.01 to 0.08)	−0.06 (−0.14 to 0.03)	−0.10 (−0.19 to 0.01)	0.19 (0.04 to 0.34)
***Gestational hypertension***					
White British women	0	0	0	0	0
Pakistani women	−1.58 (−3.60 to 0.44)	0.12 (−0.05 to 0.29)	−0.21 (−0.59 to 0.16)	−0.04 (−0.35 to 0.43)	0.29 (−0.34 to 0.93)
***Pre-eclampsia***					
White British women	0	0	0	0	0
Pakistani women	0.48 (−2.93 to 3.88)	−0.16 (−0.45 to 0.13)	0.86 (0.28 to 1.45)	−0.32 (−0.98 to 0.34)	0.77 (0.41 to 1.96)

Adjusted model adjusted for time to delivery and for maternal pregnancy booking BMI, age, parity, smoking in pregnancy status, education, gestational diabetes and infant gender.

In reference category: No hypertensive disorder (white British women) mean DBP at 8 wk (mm Hg) 62.31 (61.21 to 63.40); mean DBP change (mm Hg/wk): 8–24 wk 0.02 (−0.08 to 0.11); 24–30 wk 0.25 (0.06 to 0.44); 30–36 wk 0.34 (0.15 to 0.54); >36 wk 1.28 (0.96 to 1.59).

In reference category: gestational hypertension (white British women), mean DBP at 8 wk (mm Hg) 64.84 (60.88 to 68.80); mean DBP change (mm Hg/wk): 8–24 wk 0.02(−0.31 to 0.36); 24–30 wk 1.07 (0.35 to 1.79); 30–36 wk 0.15 (−0.59 to 0.89); >36 wk 3.04 (1.93 to 4.15).

In reference category: pre-eclampsia (white British women), mean DBP at 8 wk (mm Hg) 69.38 (61.89 to 76.89); mean DBP change (mm Hg/wk): 8–24 wk −0.02 (−0.66 to 0.70); 24–30 wk −0.12.

(−1.44 to 1.20); 30–36 wk 1.90 (0.56 to 3.25); >36 wk 2.68 (0.13 to 5.24).

There was no evidence of an early pregnancy decline in SBP or DBP in normotensive women for either ethnic group, with a very slight increase in BP in both groups across pregnancy up to 24 weeks, after which the magnitude of rate of change increased (see Fig. 2a,b and Tables 4 and 5).

As with normotensive Pakistani women, Pakistani women who developed GH had lower SBP (−4.93, 95% CI: −7.74 to −2.12) and DBP (−1.58, 95% CI: −3.60 to 0.44) at the start of pregnancy than white British women who developed this condition, and continued with lower levels until 36 weeks. From 36 weeks onwards, a steeper increase in SBP occurred than the increase demonstrated by white British women, such that by delivery Pakistani women with GH had similar SBP and DBP to white British women with GH (see Fig. 2c,d and Tables 4 and 5).

Pakistani women who developed PE began pregnancy with lower SBP (−2.58, 95% CI: −7.16 to 2.00) but similar DBP (0.48, 95% CI: −2.93 to 3.88) than white British women who developed this condition. Pakistani women demonstrated a steeper increase in BP from 30 weeks gestation than white British women. (see Fig. 2e,f). The steepness of the increase continued to term, which meant that by delivery, Pakistani women’s SBP and DBP were similar to those of white British women. White British women who developed PE showed a decline in BP from 24 weeks, their BP reached a nadir at 30 weeks, after which time both SBP and DBP increased until term. Pakistani women with PE did not show a similar decline in BP.

Supplementary Figures 1a to 7b show the adjusted (Model 2) average shaped SBP trajectories and Supplementary Figures 8a to 14b the adjusted (Model 2) average shaped DBP trajectories for Pakistani and white British women by HDP category and maternal risk factor. These trajectories were consistent with what we would expect from patterns seen with associations of each risk factor with each HDP. Supplementary Tables 1 and 2 show the adjusted mean difference (with 95% CI) in SBP and DBP at eight weeks and change in SBP and DBP in each period of gestation by GH and PE for each ethnic group.

Table 6 shows the associations of BP at eight weeks and changes in BP with time to delivery. SBP and DBP at 8 weeks gestation and BP change in any period of pregnancy was not associated with gestational length in either ethnic group, with the exception of SBP, but not DBP change between 8 and 24 weeks which was weakly associated with a reduction in gestational length for white British women only.

**Table 6:** Associations of blood pressure at 8 weeks gestation and changes in blood pressure in each period of pregnancy with the time to delivery.

Blood pressure variable	Length of gestation			
	White British women		Pakistani women	
	% Increase in gestation	95% CI	% Increase in gestation	95% CI
SBP at 8 weeks, mmHg	0.00	0.00, 0.01	0.00	−0.01, 0.00
**SBP change, mmHg/wk**				
8–24 wk	−0.26	−0.74, −0.01	−0.07	−0.56, 0.28
24–30 wk	0.09	−1.80, 1.20	−0.02	−2.43, 1.14
30–36 wk	−0.54	−32.55, 2.09	−0.09	−2.15, 1.04
≥36 wk	−0.21	−1.18, 0.45	−0.09	−0.28, 0.03
DBP at 8 weeks, mmHg	0.00	0.01, 0.02	0.00	−0.01, 0.00
**DBP change, mmHg/wk**				
8–24 wk	−0.03	−4495.20, 2.27	−0.38	−2.96, 0.29
24–30 wk	−0.19	−5.51, 1.51	−0.11	−4.51, 1.44
30–36 wk	−0.42	−1.42, 0.32	−0.26	−1.36, 0.53
>36 wk	−0.08	−0.77, 0.49	−0.04	−0.43, 0.30

Model 2 adjusted for HDP category and maternal covariates and for SBP/DBP at 8 weeks’ gestation and SBP/DBP change in BP in earlier periods of pregnancy.

Supplementary Table 3 shows similar predicted lengths of gestation at 2 SDs above and below the average SBP and DBP at 8 weeks and changes in SBP and DBP in each period of gestation for Pakistani and white British women.

## Discussion

We found that Pakistani women start pregnancy with lower SBP and DBP than white British women. This is true for women who remain normotensive throughout their pregnancies and for those who go on to develop GH or PE. Generally, normotensive Pakistani and white British women, show gradual increases in BP until 36 weeks when SBP and DBP for both ethnic groups increases more steeply until term. Compared with white British women, Pakistani women who developed either GH or PE had a steeper rise in BP in the third trimester and this difference in BP trajectories influenced the comparison of HDP between the two groups. GH was more common in white British women due to: (a) the combination of the same diagnostic criteria being used across ethnicities, (b) Pakistani women having lower BP levels until the final trimester of pregnancy, and (c) women in both ethnic groups who developed GH doing so evenly between 20–36 and after 36 weeks gestation. By contrast, the proportion with PE was similar in both ethnic groups, because in both groups those developing PE were much more likely to do so after 36 weeks, which meant that the steeper third trimester rise in BP in Pakistani women had a greater impact on their PE rate than their GH rate.

A number of previous studies have reported ethnic differences in the occurrence of GH and PE and the association of risk factors with GH and PE and adverse outcomes for different ethnic groups^36,46,47^ and one study found similar rates of PE in South Asian and non-Hispanic white Americans^36^. However, few previous studies have examined ethnic differences in BP trajectories across pregnancy^38^. A recent study compared Norweigian women with women from eastern Europe, middle east, Africa and south and east Asia, however only 200 south Asian women were included and BP was recorded just twice antenatally^39^.

The lower rate of GH in Pakistani compared with white British women in our study is not surprising given the lower SBP and DBP of Pakistani women at the start of pregnancy and between 20 and 36 weeks gestation, and that criteria for the diagnosis of HDP are the same in both ethnic groups^22^.

Our findings provide some evidence for considering the rate of change or trajectory patterns of gestational BP, particularly for understanding ethnic differences in rates of HDP. The use of BP change across pregnancy (usually an increase of >15 mmHg or more from baseline reading) to identify women at increased risk of an adverse outcome has been explored for over 30 years^48^. However inconsistencies in study methods and their reported findings, together with the complexity of incorporating such measures into clinical guidelines and practice has so far prevented its adoption^49,50^.

We did not show a decline in BP from early pregnancy to 18/20 weeks that previous studies found^18,38,51^. This is unlikely to reflect a lack of measurements at 18 to 24 weeks because antenatal measurements were obtained at regular intervals across pregnancy and this did not change over the period of the study^22,41^. The transitory decline in BP in early pregnancy is thought to reflect the early haemodynamic changes of pregnancy and its end point (around 20 weeks) is used as the marker after which GH and PE are diagnosed. Our finding of a gradual increase in BP in the first two trimesters of pregnancy for normotensive women and women with GH and PE (with the exception of white British women with PE, who showed a decline from 24 to 30 weeks), in both ethnic groups, may reflect the changing characteristics of pregnant women over time and the greater prevalence of risk factors for HDP^19^. For example, the proportion of women who were overweight and obese included in our previous UK cohort which recruited women in pregnancy in 1990 were 15% and 6%, respectively^18,52^; in this study, which recruited women 20 years later and from an area of the UK with more deprivation, 29% were overweight and 22% obese^40^.

Our study is large with many repeated measurements, which allowed a consistent approach to the classification of GH and PE. Use of routinely collected data will introduce a degree of variability, for example use of different BP measurement devices or use of different sized arm cuffs, however all measurements were collected in accordance with clinical care at the time of recruitment and accurately reflect the measurements used to inform clinical decisions related to the diagnosis and treatment of HDP in our study population.

The most recent International Society for the Study of Hypertension in pregnancy (ISSHP) guidance defines PE as high BP on at least two occasions after 20 weeks with concurrent significant proteinuria defined as 2+ on a reagent strip^21^, the UK NICE do not comment on how many + are appropriate^22^. Our use of 1+ proteinuria on reagent strip in defining PE reflects the ISSHP criteria for diagnosing PE and what was used in clinical practice in the UK at the time of our study (which preceded the most recent ISSHP guidelines)^53^.

Compared with the most recent ISSHP criteria our use of 1+ proteinuria may have increased the overall prevalence of PE in our study. However, this will not have altered the ethnic differences in BP trajectories nor the similarity in PE between white British and Pakistani women, which was driven by the steepear increase in BP in Pakistani women. Some of the weaker associations of risk factors with PE compared with GH shown in Table 3 may be because of a lower proteinuria threshold, but given the relatively small numbers with PE and imprecise estimates with wide confidence intervals, we would not be able to distinguish differences in associations with a stricter definition of proteinuria, as the number with PE would be smaller.

Other measures of proteinuria, for example protein-creatinine ratio, may more accurately estimate proteinuria, but they were not consistently recorded at each antenatal visit. Indicators of severe PE, such as evidence of multi-organ involvement were unavailable in our study, so we cannot assess severe PE or eclampsia.

In conclusion, BP trajectories differ by ethnicity. Pakistani women had lower BP at the start of pregnancy and during the first two trimesters, irrespective of the development of a HDP or the presence of a risk factor. Compared to white British women, Pakistani women who developed GH or PE showed steeper increases in BP towards the end of pregnancy. Because GH developed evenly between 20–36 weeks and after 36 weeks gestation and PE occurred more commonly after 36 weeks in both ethnic groups, lower BP up to the third trimester in Pakistani women resulted in a considerably lower GH rate, whereas PE rate, influence by the steep third trimester BP increase is similar in the two groups. Criteria for diagnosing HDP may benefit from considering ethnic differences in BP change across pregnancy.

## Supplementary information

Supplementary Tables and Figures

## Data Availability

The Born in Bradford Study allows bone fide researchers to access data. Full details of how to do this are provided on the study web pages (https://borninbradford.nhs.uk/research/how-to-access-data/).
