# Supplementary material for: Blood pressure change across pregnancy in white British and Pakistani women: analysis of data from the Born in Bradford cohort
Source: Sci Rep. 2019 Sep 13;9:13199. doi: 10.1038/s41598-019-49722-9 (PMC6744423; doi:10.1038/s41598-019-49722-9)

Blood pressure change across pregnancy in white British and Pakistani women: analysis of data from the Born in Bradford cohort

Diane Farrar, Gillian Santorelli, Debbie A Lawlor, Derek Tuffnell, Trevor A Sheldon, Jane West, Corrie Macdonald-Wallis

Supplementary Table 1 Adjusted mean difference (95% CI) in SBP at eight weeks and change in systolic blood pressure in each period of gestation by hypertensive disorders of pregnancy (no HDP, gestational hypertension and preeclampsia)

		**Mean difference in average SBP change, mmHg/wk**			
Hypertensive disorder	Mean Difference in SBP at 8 wk, mmHg	8-24 weeks	24-30 weeks	30-36 weeks	≥36 weeks
White British women					
No HDP	0	0	0	0	0
Gestational ht	1.75 (-0.90 to 4.41)	0.11 (-0.11 to 0.32)	0.19 -0.24 to 0.63)	0.35 (-0.08 to 0.77)	-0.11 (-0.78 to 0.55)
Preeclampsia	-3.63 (-5.87 to -1.40)	-0.12 (-0.31 to 0.06)	0.28 -0.09 to 0.66)	-0.36 (-0.72 to 0.01)	-1.20 (-1.76 to -0.64)
Pakistani women					
No HDP	0	0	0	0	0
Gestational ht	1.19 (-2.24 to 4.63)	-0.06 (-0.33 to 0.21)	0.26 (-0.27 to 0.80)	0.93 (0.38 to 1.48)	0.94 (0.03 to 1.87)
Preeclampsia	-4.33 (-7.19 to -1.49)	0.25 (-0.47 to -0.01)	0.11(-0.33 to 0.55)	-0.32 (-0.76 to 0.11)	-0.35 (-1.06 to 0.35)

HDP= hypertensive disease of pregnancy

Adjusted for time to delivery and for maternal pregnancy booking BMI, age, parity, smoking in pregnancy status, education, gestational diabetes and infant gender. Reference category = women with no HDP.

In reference category: white British women (normotensive women), mean SBP at 8 wk (mm Hg) 112.45 (107.84 to 117.07); mean SBP change (mm Hg/wk): 8–24 wk 0.40 (0.01 to 0.78); 24–30 wk -0.37 (-1.14 to 0.41); 30–35[6] wk 0.89 (0.13 to 1.64); >36 wk 3.67 (2.51 to 4.82)

In reference category: Pakistani women (normotensive women), mean SBP at 8 wk (mm Hg) 110.46 (104.65 to 116.26) mean SBP change (mm Hg/wk): 8–24 wk 0.65 (0.19 to 1.12); 24–30 wk -0.07 (-0.97 to 0.83); 30–36 wk 0.85 (-0.06 to 1.74); >36 wk 2.09 (0.66 to 3.52)

Supplementary Table 2 Adjusted mean difference (95% CI) in DBP at eight weeks and change in diastolic blood pressure in each period of gestation by hypertensive disorders of pregnancy (no HDP (reference), gestational hypertension and preeclampsia)

		**Mean difference in average DBP change, mmHg/wk**			
Hypertensive disorder	Mean Difference in DBP at 8 wk, mmHg	8-24 weeks	24-30 weeks	30-36 weeks	≥36 weeks
White British women					
No HDP	0	0	0	0	0
Gestational ht	1.62 (-0.27 to 0.30)	0.13 (-0.04 to 0.28)	-0.29 (-0.05 to 0.63)	0.07 (-0.28 to 3.54)	0.19 (-0.36 to 0.7)
Preeclampsia	-2.29 (-3.89 to -0.68)	0.00 (-0.13 to 0.14)	-0.27 (-0.02 to 0.56)	-0.40 (-0.70 to -0.10)	-0.43 (-0.89 to 0.02)
Pakistani women					
No HDP	0	0	0	0	0
Gestational ht	3.11 (0.59 to 5.63)	-0.04 (-0.25 to 0.16)	0.63 (0.22 to 1.03)	0.46 (0.04 to 0.87)	0.51 (-0.22 to 1.25)
Preeclampsia	-1.32 (-3.41 to 0.76)	-0.12 (-0.29 to 0.05)	0.20 (-0.13 to 0.53)	-0.27 (-0.61 to 0.06)	-0.64 (-1.21 to -0.08)

HDP= hypertensive disease of pregnancy

Adjusted for time to delivery and for maternal pregnancy booking BMI, age, parity, smoking in pregnancy status, education, gestational diabetes andgender. Reference category = women without a HDP.

In reference category: white British women (normotensive women), mean DBP at 8 wk (mm Hg) 66.594 (63.27 to 69.90) mean DBP change (mm Hg/wk): 8–24 wk 0.01 (-0.27 to 0.30); 24–30 wk -0.26 (-0.86 to 0.33); 30–36 wk 1.14 (0.54 to 1.75); >36 wk 2.21 (1.27 to 3.16)

In reference category: Pakistani women (normotensive women), mean DBP at 8 wk (mm Hg) 62.14 (57.88 to 66.39) mean DBP change (mm Hg/wk): 8–24 wk 0.38 (0.03 to 0.72); 24–30 wk -0.20 (-0.87 to 0.47); 30–36 wk 0.83 (0.15 to 1.52); >36 wk 2.69 (1.55 to 3.84)

Supplementary Table 3 Average length of gestation by standard deviations of blood pressure at eight weeks gestation and change in blood pressure in each period of pregnancy in the joint model adjusted for maternal covariates

Blood pressure variable	**Length of gestation**	
	Average length of gestation	95% CI
	**White British women**	
Average SBP and DBP at 8 weeks and average change in SBP and DBP in each period of gestation		
SBP at 8 weeks (1 SD = 7.34mm Hg)		
+ 2 SD	39.87	39.70, 40.04
- 2 SD	39.70	39.50, 39.85
SBP change 8-24 weeks (1 SD = 0.32mm Hg/week)		
+ 2 SD	39.40	38.74, 39.76
- 2 SD	39.99	39.61,40.67
SBP change 24-30 weeks (1 SD =0.55mm Hg/week)		
+ 2 SD	40.01	35.03, 42.72
- 2 SD	39.52	33.04, 42.26
SBP change 30-36 weeks (1 SD = 0.66mm Hg/week)		
+ 2 SD	38.08	-147.72, 44.82
- 2 SD	41.06	-106.69, 44.86
SBP change >36 weeks (1 SD = 1.03 mm Hg/week)		
+ 2 SD	38.74	32.67, 41.67
- 2 SD	40.64	36.78, 42.78
DBP at 8 weeks (1 SD = 4.53mm Hg)		
+ 2 SD	39.91	39.69, 40.13
- 2 SD	39.64	39.40, 39.85
DBP change 8-24 weeks (1 SD = 0.12mm Hg/week)		
+ 2 SD	39.26	10.47, 44.13
- 2 SD	40.24	13.51, 44.21
DBP change 24-30 weeks (1 SD =0.39mm Hg/week)		
+ 2 SD	39.43	31.33, 42.77
- 2 SD	40.09	32.78, 43.00
DBP change 30-36 weeks (1 SD = 0.66mm Hg/week)		
+ 2 SD	39.16	35.90, 41.70
- 2 SD	40.33	38.63, 42.24
DBP change >36 weeks (1 SD = 0.95 mm Hg/week)		
+ 2 SD	39.42	35.89, 41.70
- 2 SD	40.11	36.72, 42.00
	**Pakistani women**	
SBP at 8 weeks (1 SD =6.99mm Hg)		
+ 2 SD	39.53	39.37, 39.69

- 2 SD	39.73	39.34, 39.88
SBP change 8-24 weeks (1 SD =0.29mm Hg/week)		
+ 2 SD	39.54	38.90, 40.04
- 2 SD	39.73	39.20, 40.28
SBP change 24-30 weeks (1 SD =0.59mm Hg/week)		
+ 2 SD	39.58	36.77, 42.62
- 2 SD	39.52	32.88, 41.50
SBP change 30-36 weeks (1 SD = 0.71mm Hg/week)		
+ 2 SD	39.34	31.16, 42.75
- 2 SD	39.92	32.18, 42.92
SBP change >36 weeks (1 SD = 1.23mm Hg/week)		
+ 2 SD	39.09	36.27, 40.94
- 2 SD	40.13	37.89, 41.70
DBP at 8 weeks (1 SD = 5.04mm Hg)		
+ 2 SD	39.52	39.34, 39.70
- 2 SD	39.74	39.57, 39.91
DBP change 8-24 weeks (1 SD = 0.22mm Hg/week)		
+ 2 SD	39.25	37.28, 39.94
- 2 SD	39.99	39.31, 40.27
DBP change 24-30 weeks (1 SD =0.34mm Hg/week)		
+ 2 SD	39.45	33.65, 42.30
- 2 SD	39.80	34.33, 42.46
DBP change 30-36 weeks (1 SD = 0.57 mm Hg/week)		
+ 2 SD	39.30	35.83, 41.03
- 2 SD	39.95	37.74, 41.86
DBP change >36 weeks (1 SD = 1.11 mm Hg/week)		
+ 2 SD	39.42	37.12, 41.06
- 2 SD	39.84	37.69, 41.35

Predictions are for women in the reference category: women without a HDP, and for average levels of each of the blood pressure variables other than that being varied

Supplementary Figure 1a Predicted trajectories of systolic blood pressure across pregnancy for Pakistani women by maternal early-pregnancy BMI and hypertensive disorder. All trajectories are predicted by the fully adjusted joint model (model 2)

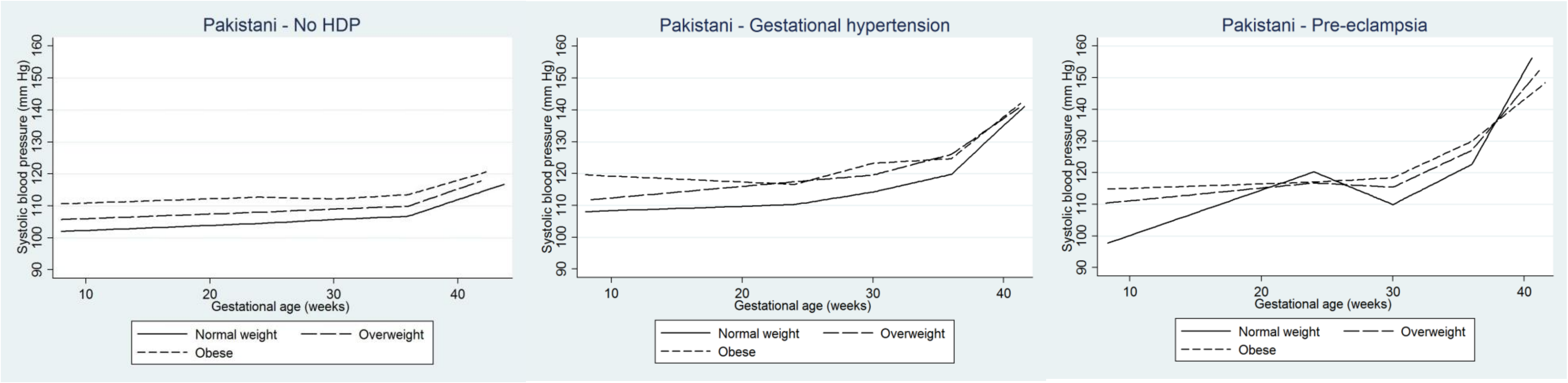

Supplementary Figure 1b Predicted trajectories of systolic blood pressure across pregnancy for white British women by maternal early-pregnancy BMI and hypertensive disorder of pregnancy

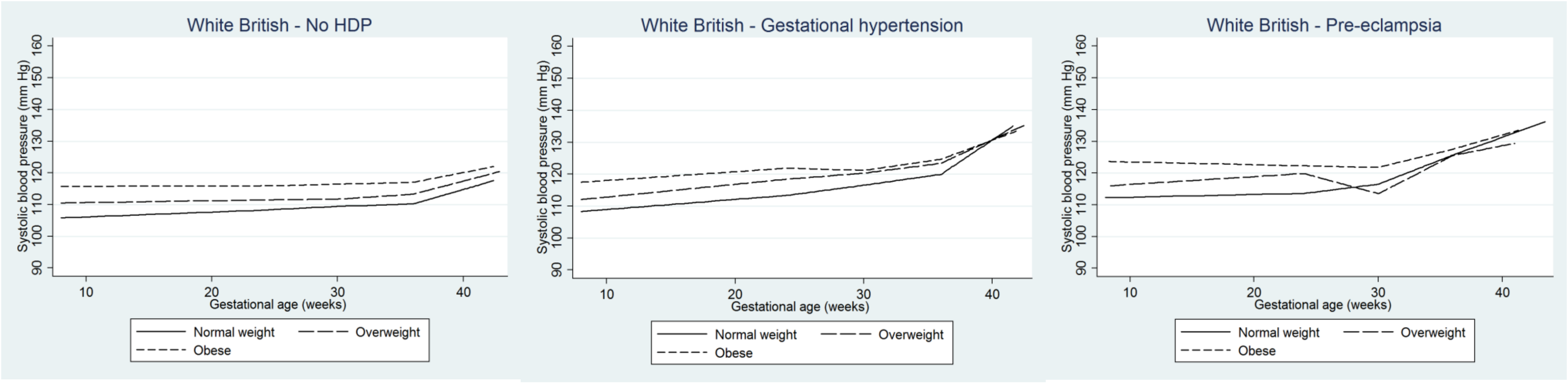

Supplementary Figure 2a Predicted trajectories of systolic blood pressure across pregnancy for Pakistani women by maternal age and hypertensive disorder of pregnancy

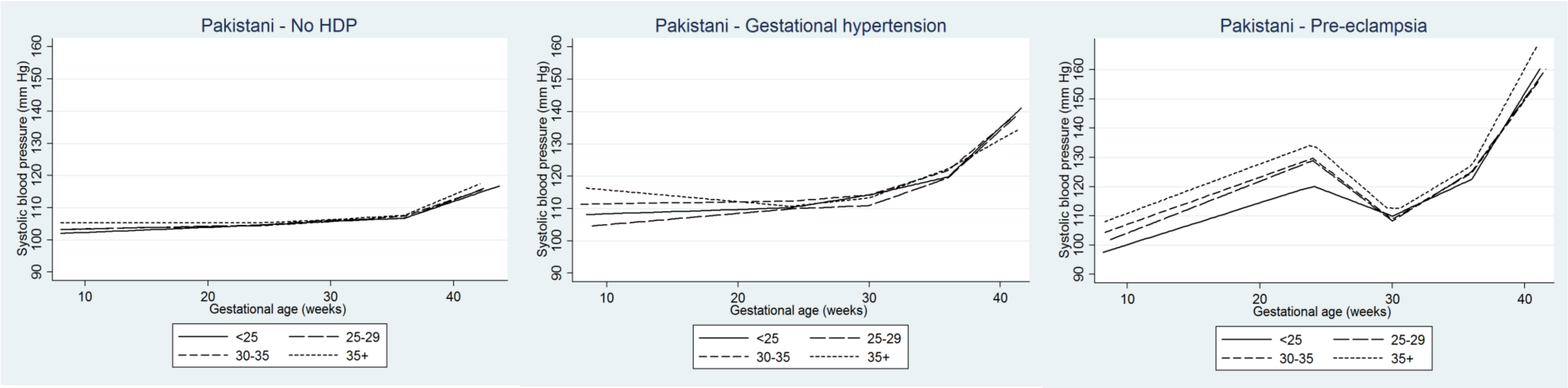

Supplementary Figure 2b Predicted trajectories of systolic blood pressure across pregnancy for white British women by maternal age and hypertensive disorder of pregnancy

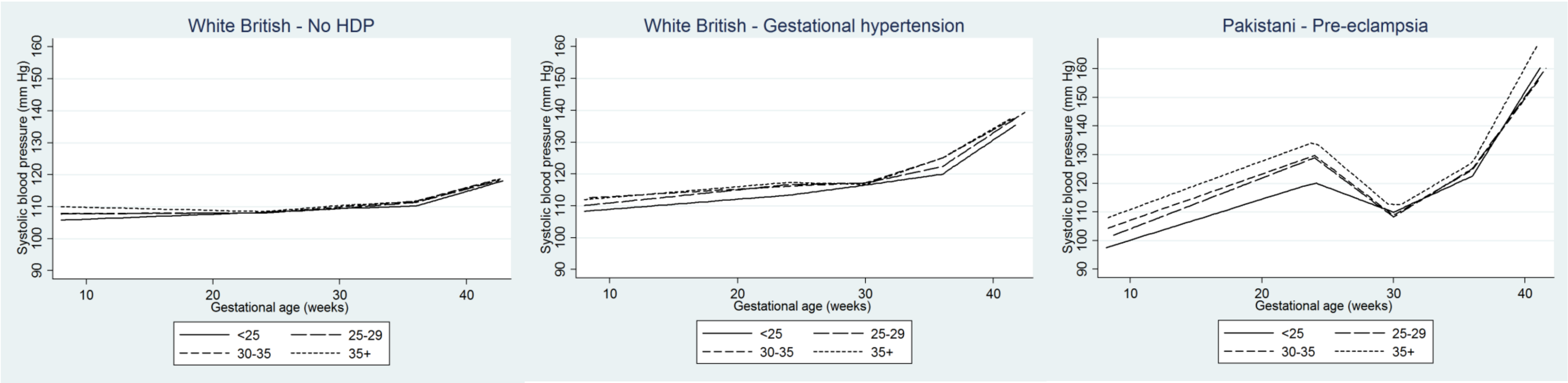

Supplementary Figure 3a Predicted trajectories of systolic blood pressure across pregnancy for Pakistani women by parity and hypertensive disorder of pregnancy

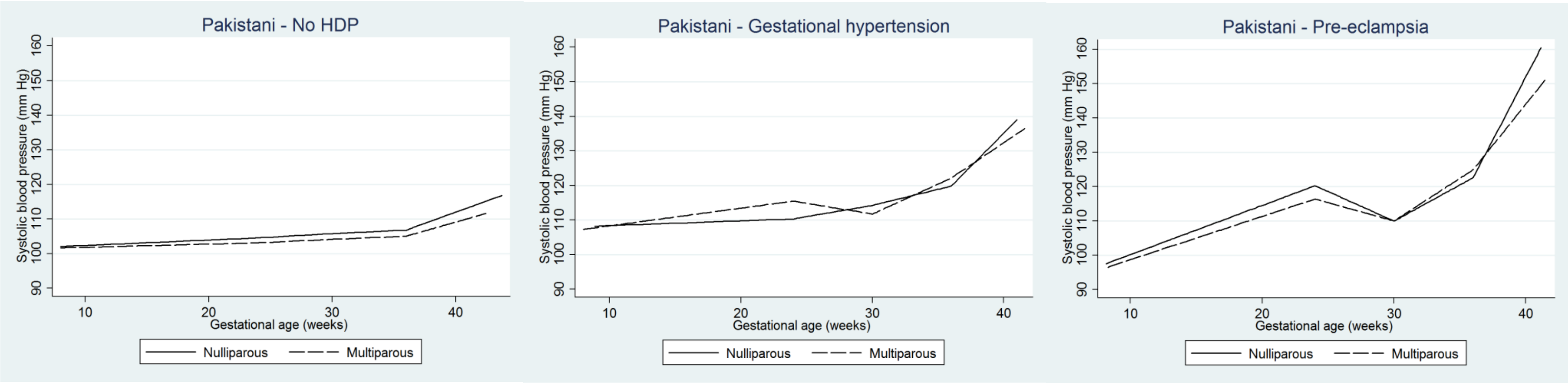

Supplementary Figure 3b Predicted trajectories of systolic blood pressure across pregnancy for white British women by parity and hypertensive disorder of pregnancy

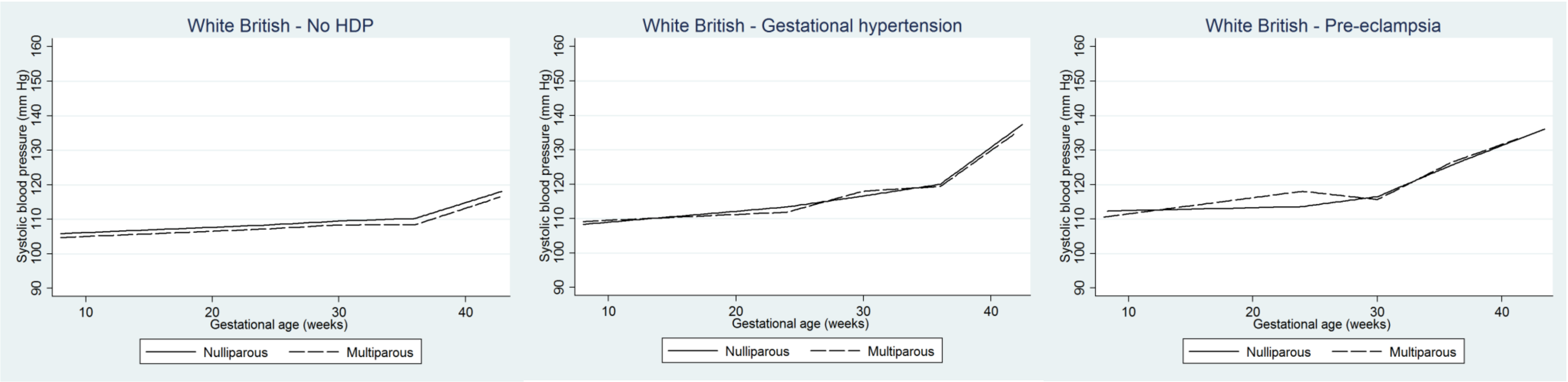

Supplementary Figure 4a Predicted trajectories of systolic blood pressure across pregnancy for Pakistani women by gestational diabetes and hypertensive disorder of pregnancy

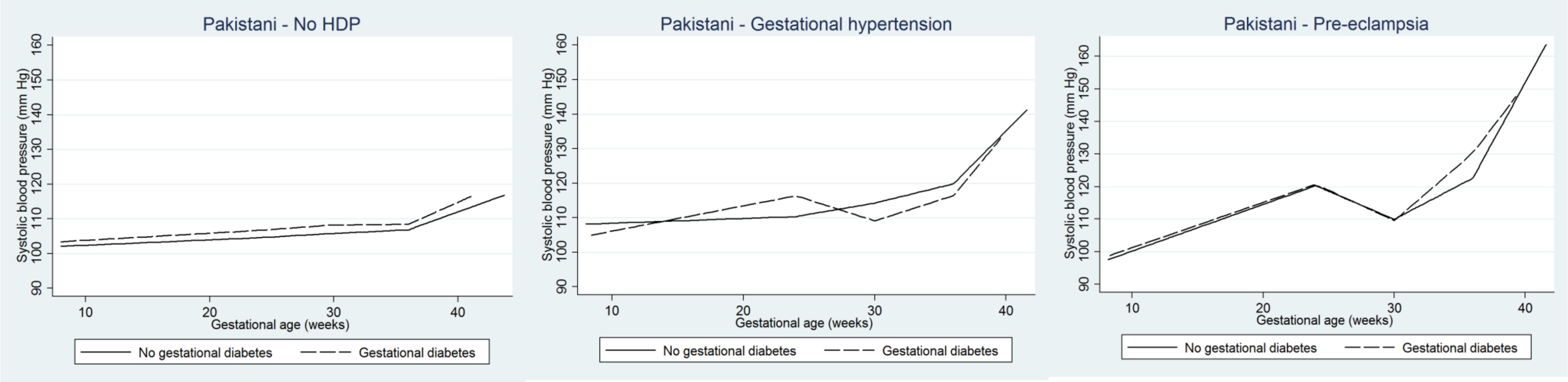

Supplementary Figure 4b Predicted trajectories of systolic blood pressure across pregnancy for white British women by gestational diabetes and hypertensive disorder of pregnancy

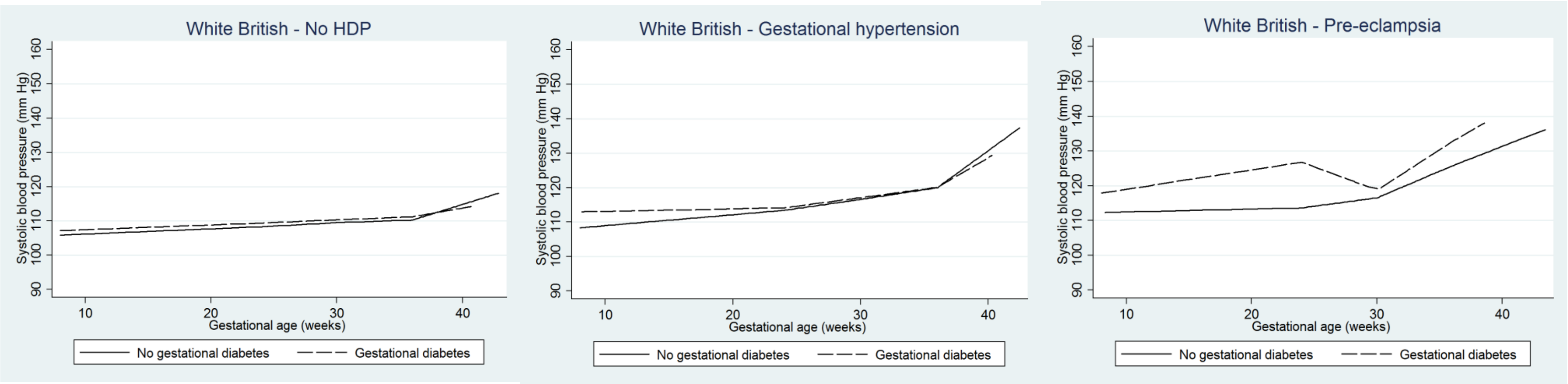

Supplementary Figure 5a Predicted trajectories of systolic blood pressure across pregnancy for Pakistani women by maternal education and hypertensive disorder of pregnancy

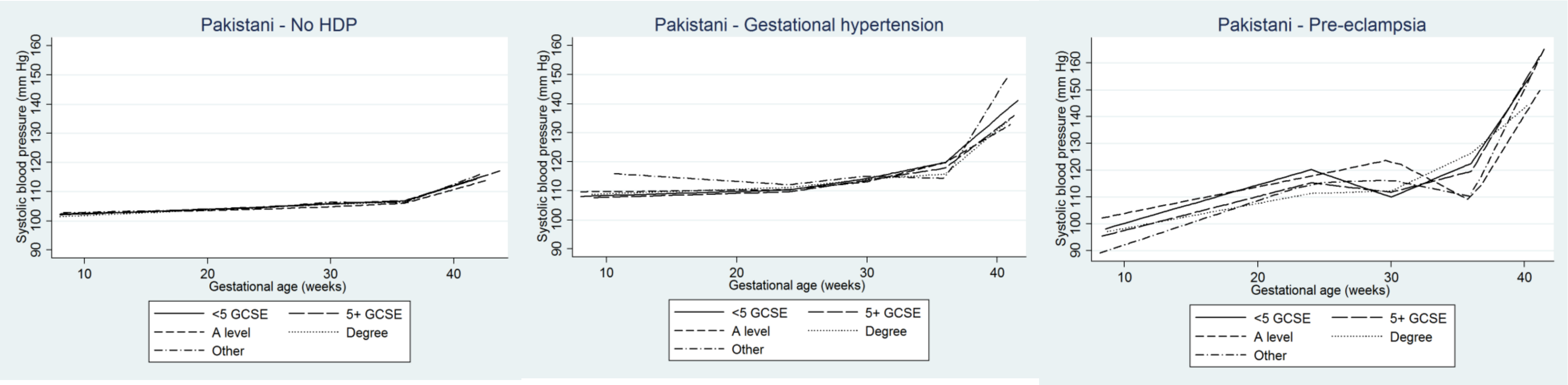

Supplementary Figure 5b Predicted trajectories of systolic blood pressure across pregnancy for white British women by maternal education and hypertensive disorder of pregnancy

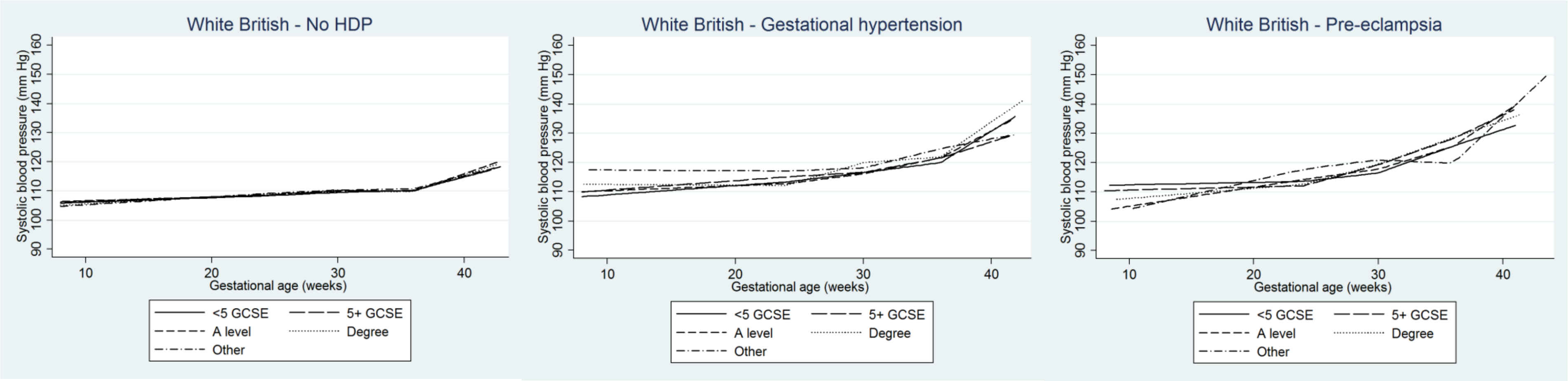

Supplementary Figure 6a Predicted trajectories of systolic blood pressure across pregnancy for white British women by smoking during pregnancy and hypertensive disorder of pregnancy

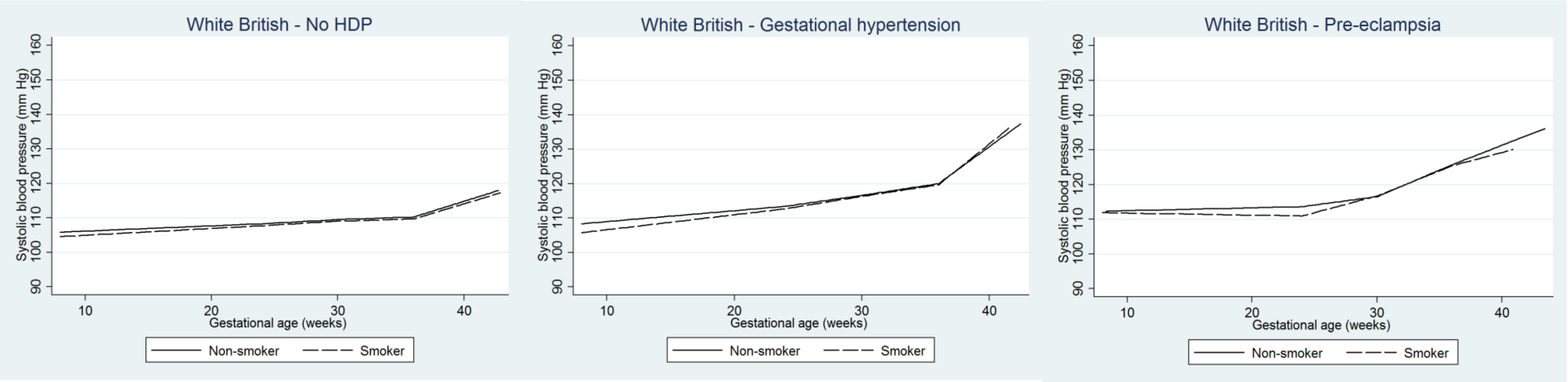

Too few Pakistani women smoked during pregnancy to be able to produce trajectories

Supplementary Figure 7a Predicted trajectories of systolic blood pressure across pregnancy for Pakistani women by infant gender and hypertensive disorder of pregnancy

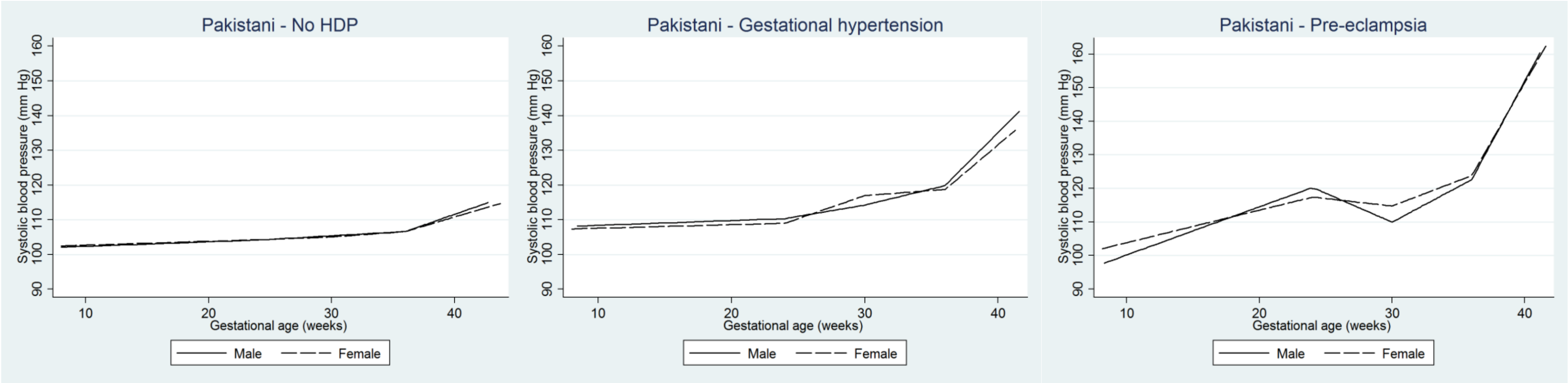

Supplementary Figure 7b Predicted trajectories of systolic blood pressure across pregnancy for white British women by infant gender and hypertensive disorder of pregnancy

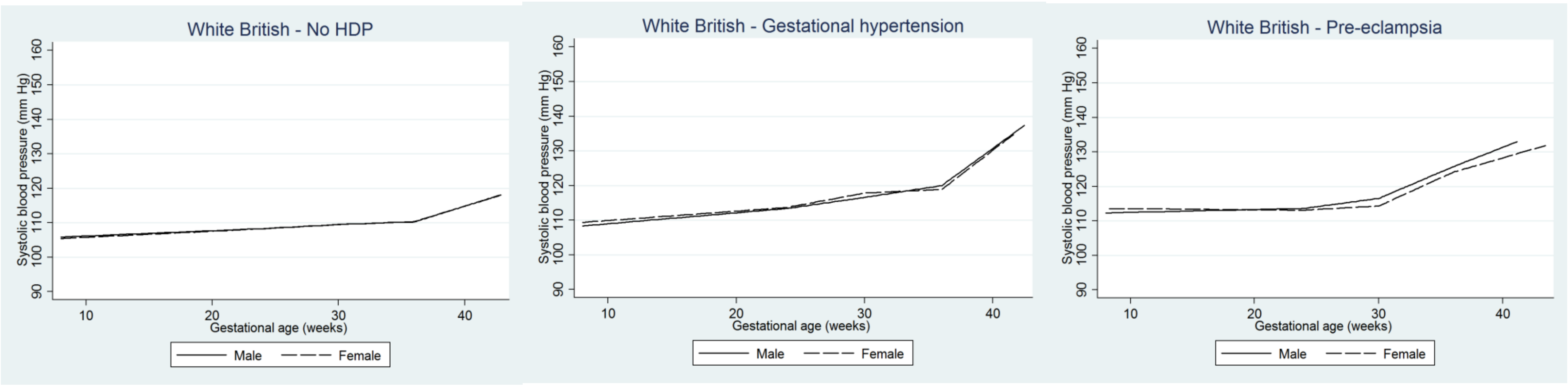

Supplementary Figure 8a Predicted trajectories of diastolic blood pressure across pregnancy for Pakistani women by maternal early-pregnancy BMI and hypertensive disorder of pregnancy

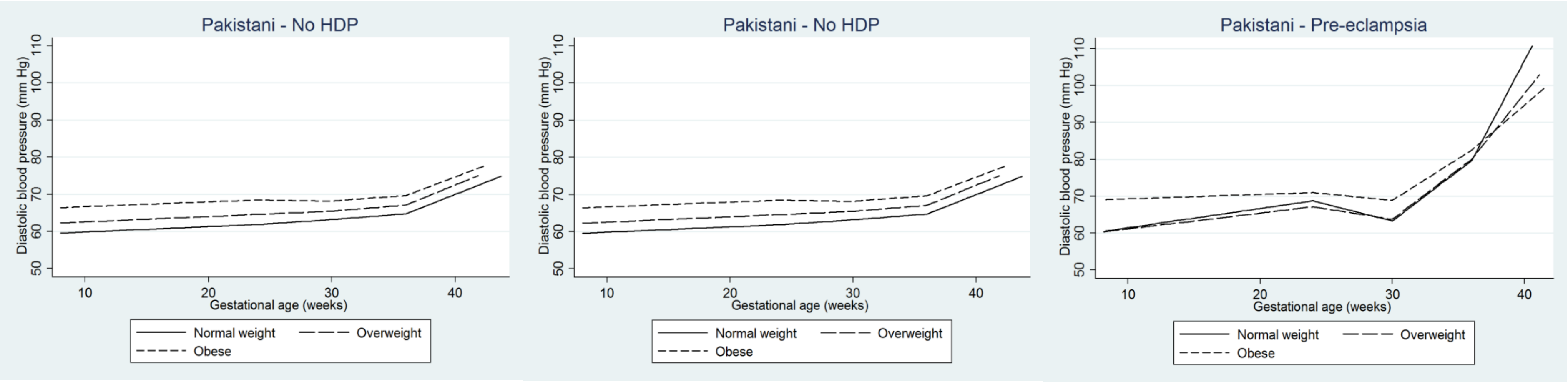

Supplementary Figure 8b Predicted trajectories of diastolic blood pressure across pregnancy for white British women by maternal early-pregnancy BMI and hypertensive disorder of pregnancy

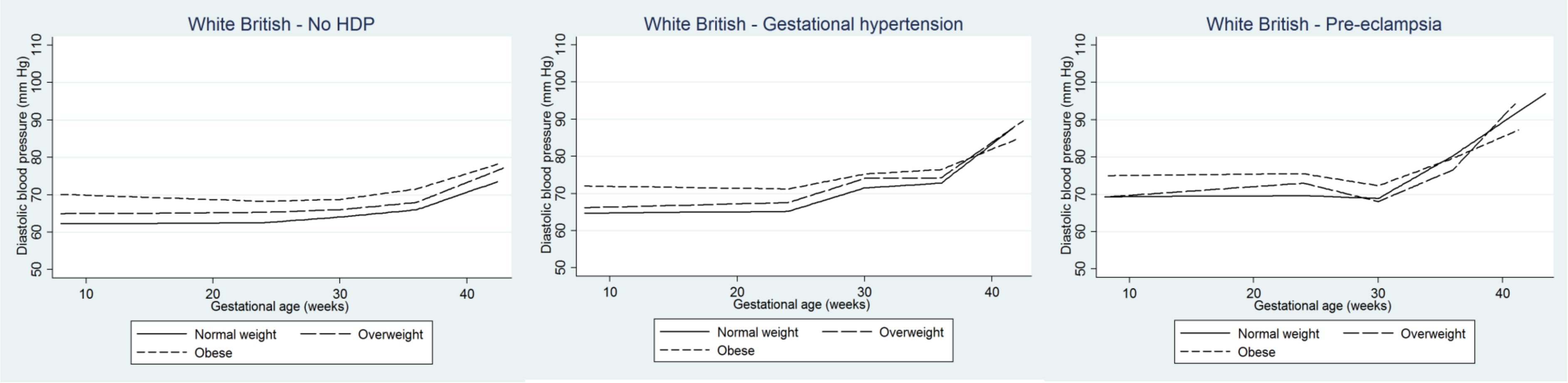

Supplementary Figure 9a Predicted trajectories of diastolic blood pressure across pregnancy for Pakistani women by maternal age and hypertensive disorder of pregnancy order of pregnancy

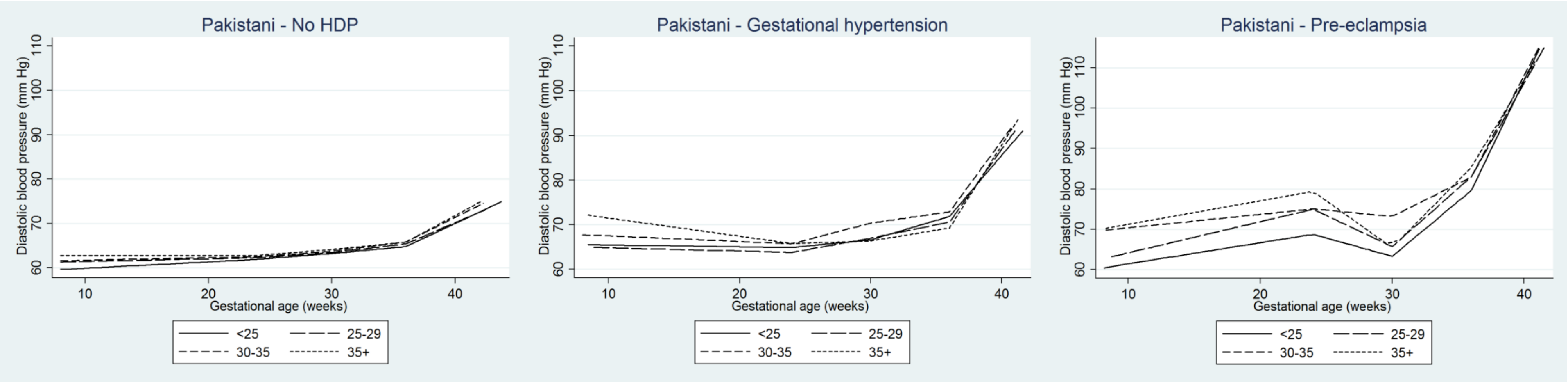

Supplementary Figure 9b Predicted trajectories of diastolic blood pressure across pregnancy for white British women by maternal age and hypertensive disorder of pregnancy order of pregnancy

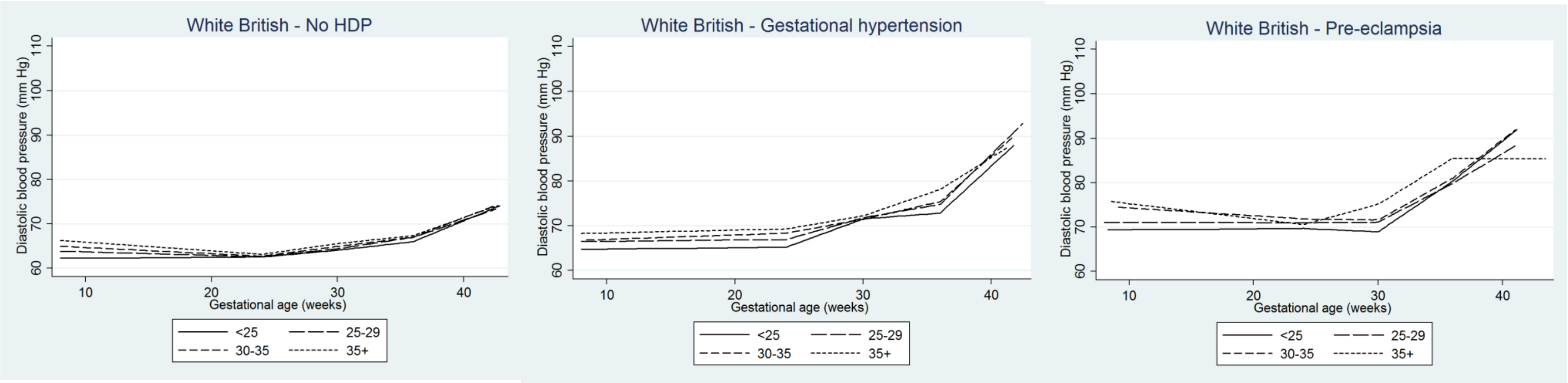

Supplementary Figure 10a Predicted trajectories of diastolic blood pressure across pregnancy for Pakistani women by parity and hypertensive disorder of pregnancy

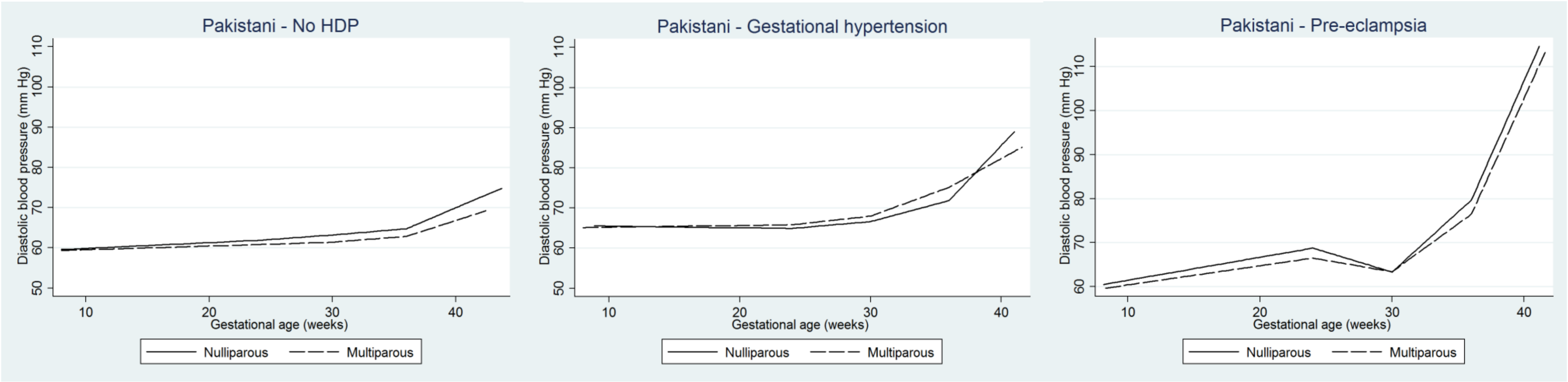

Supplementary Figure 10b Predicted trajectories of diastolic blood pressure across pregnancy for white British women by parity and hypertensive disorder of pregnancy

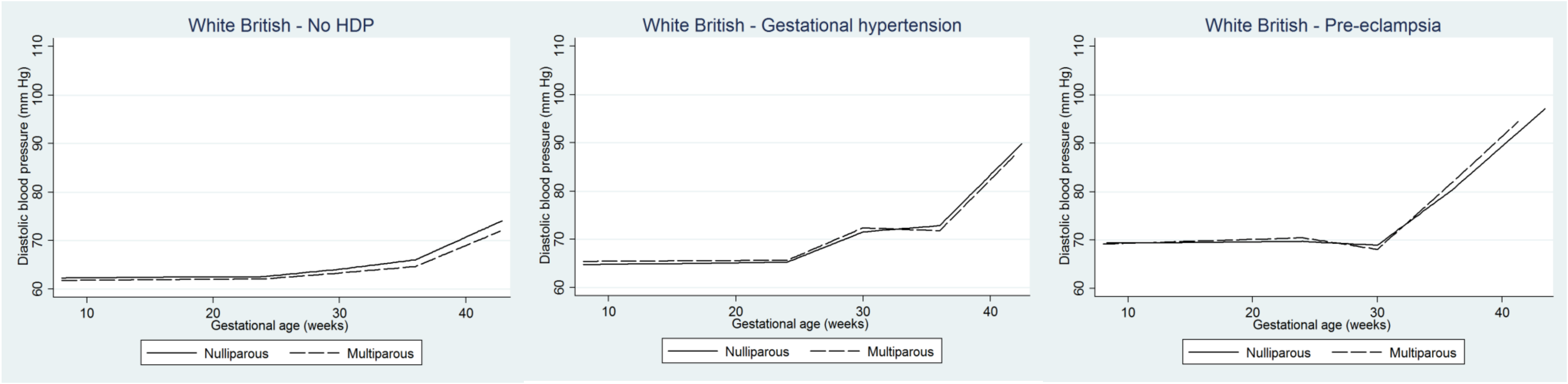

Supplementary Figure 11a Predicted trajectories of diastolic blood pressure across pregnancy for Pakistani women by gestational diabetes and hypertensive disorder of pregnancy

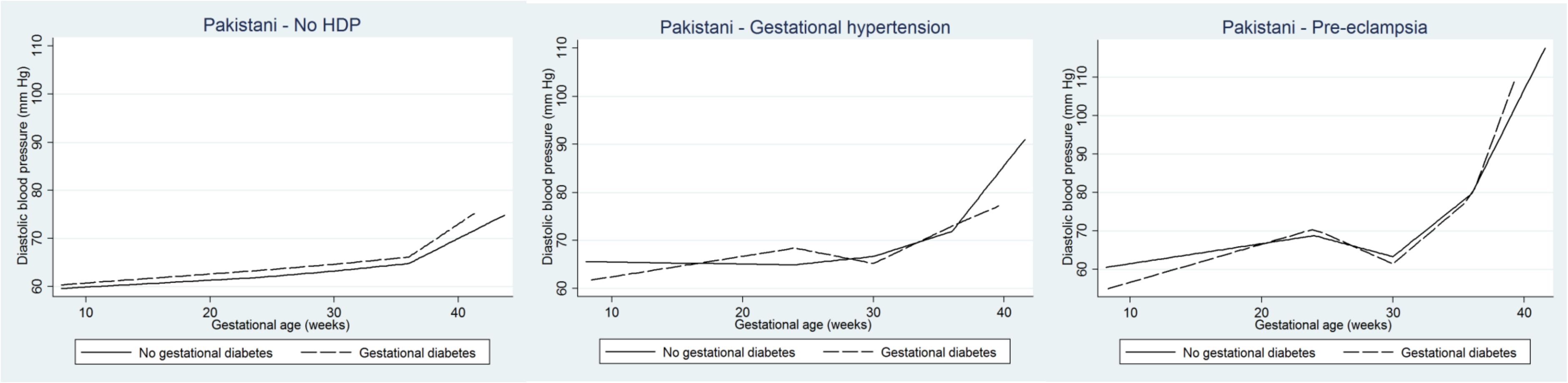

Supplementary Figure 11b Predicted trajectories of diastolic blood pressure across pregnancy for white British women by gestational diabetes and hypertensive disorder of pregnancy

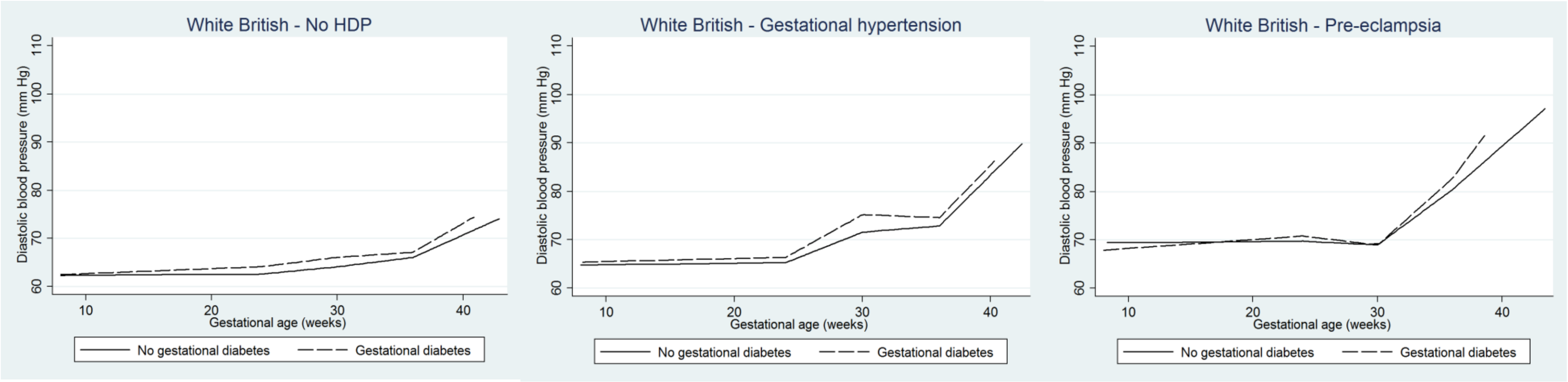

Supplementary Figure 12a Predicted trajectories of diastolic blood pressure across pregnancy for Pakistani women by maternal education and hypertensive disorder of pregnancy

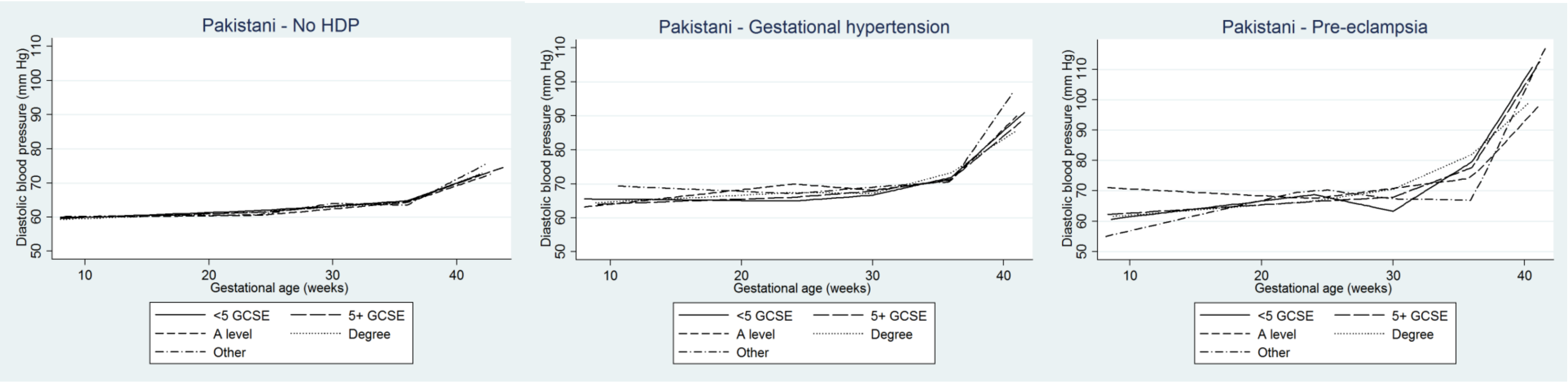

Supplementary Figure 12b Predicted trajectories of diastolic blood pressure across pregnancy for white British women by maternal education and hypertensive disorder of pregnancy

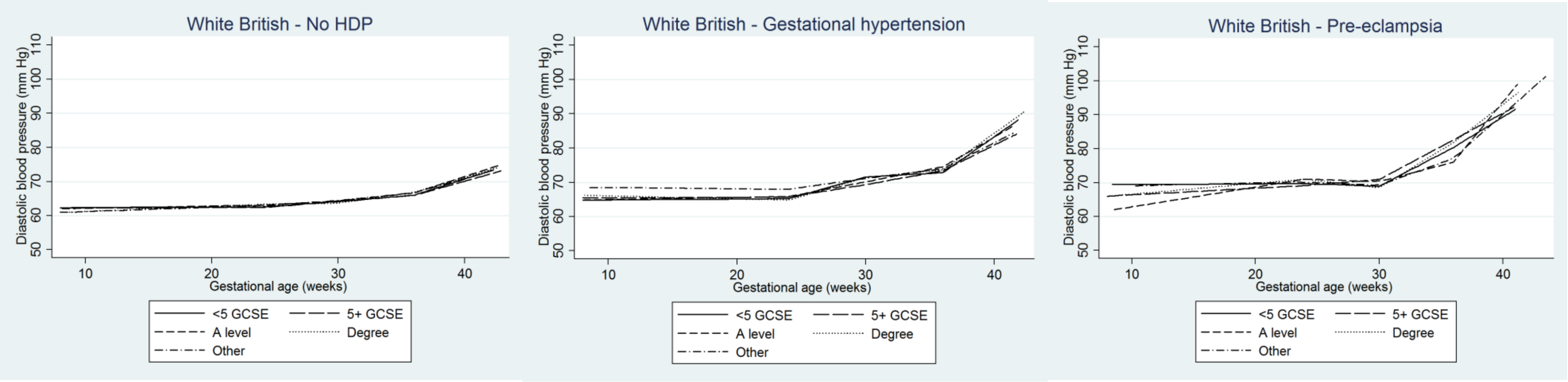

Supplementary Figure 13a Predicted trajectories of diastolic blood pressure across pregnancy for white British women by smoking during pregnancy and hypertensive disorder of pregnancy

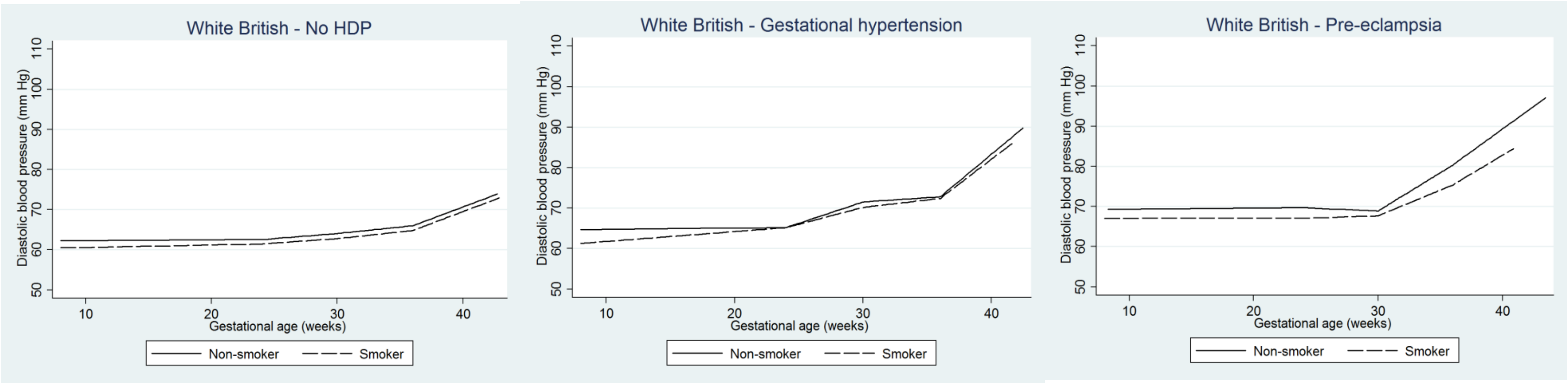

Too few Pakistani women smoked during pregnancy to be able to produce trajectories

Supplementary Figure 14a Predicted trajectories of diastolic blood pressure across pregnancy for Pakistani women by infant gender and hypertensive disorder of pregnancy

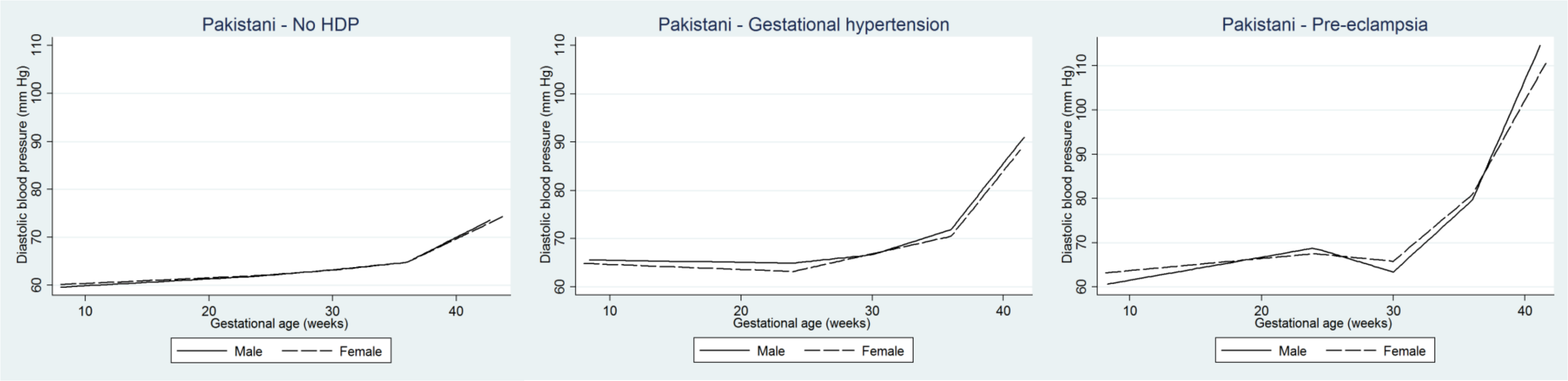

Supplementary Figure 14b Predicted trajectories of diastolic blood pressure across pregnancy for white British women by infant gender and hypertensive disorder of pregnancy

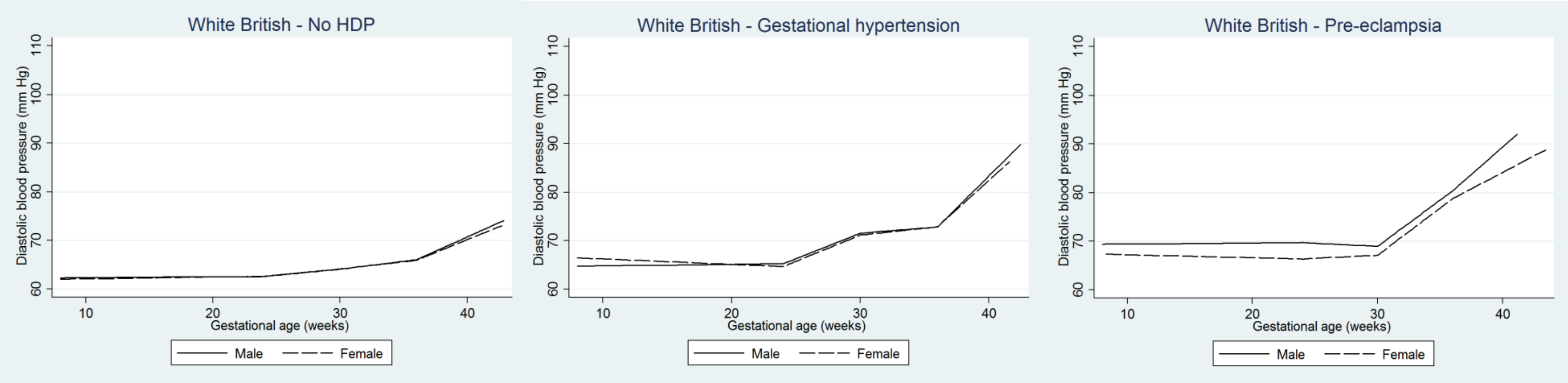

Supplement: Supplementary file 1 — Supplementary Tables and Figures [file 41598_2019_49722_MOESM1_ESM.pdf]
